# Low Incidence of Renal Dysfunction among HIV-Infected Patients on a Tenofovir-Based First Line Antiretroviral Treatment Regimen in Myanmar

**DOI:** 10.1371/journal.pone.0135188

**Published:** 2015-08-24

**Authors:** Nang Thu Thu Kyaw, Anthony D. Harries, Palanivel Chinnakali, Annick Antierens, Kyi Pyar Soe, Mike Woodman, Mrinalini Das, Sharmila Shetty, Moe Khine Lwin Zuu, Pyae Sone Htwe, Marcelo Fernandez

**Affiliations:** 1 Médecins Sans Frontières- Switzerland, Yangon, Myanmar; 2 International Union against Tuberculosis and Lung Disease, Paris, France; 3 London School of Hygiene and Tropical Medicine, London, United Kingdom; 4 Jawaharlal Institute of Postgraduate Medical Education and Research (JIPMER), Puducherry, India; 5 Médecins Sans Frontières, Bruxelles, Belgium; 6 Médecins Sans Frontières, Geneva, Switzerland; 7 Médecins Sans Frontières/Doctors Without Borders, New Delhi, India; University of Athens, Medical School, GREECE

## Abstract

**Background:**

Since 2004, Médecins Sans Frontières-Switzerland has provided treatment and care for people living with HIV in Dawei, Myanmar. Renal function is routinely monitored in patients on tenofovir (TDF)-based antiretroviral treatment (ART), and this provides an opportunity to measure incidence and risk factors for renal dysfunction.

**Methods:**

We used routinely collected program data on all patients aged ≥15 years starting first-line TDF-based ART between January 2012 and December 2013. Creatinine clearance (CrCl) was assessed at base line and six-monthly, with renal dysfunction defined as CrCl < 50ml/min/1.73m^2^. We calculated incidence of renal dysfunction and used Cox regression analysis to identify associated risk factors.

**Results:**

There were 1391 patients, of whom 1372 had normal renal function at baseline. Of these, 86 (6.3%) developed renal dysfunction during a median time of follow-up 1.14 years with an incidence rate of 5.4 per 100 person-years: 78 had CrCl between 30–50ml/min/1.73m^2^ and were maintained on TDF–based ART, but 5 were changed to another regimen: 4 because of CrCl <30ml/min/1.73m^2^. Risk factors for renal dysfunction included age ≥45 years, diagnosed diabetes, underlying renal disease, underweight and CD4 count <200cells/mm^3^. There were 19 patients with baseline renal dysfunction and all continued on TDF-based ART: CrCl stayed between 30–49 ml/min/1.73m^2^ in five patients while the remainder regained normal renal function.

**Conclusions:**

In a resource-poor country like Myanmar, the low incidence of renal toxicity in our patient cohort suggests that routine assessment of CrCl may not be needed and could be targeted to high risk groups if resources permit.

## Introduction

HIV/AIDS has been transformed from a short-lived fatal disease to a chronic manageable disease through the use of antiretroviral therapy (ART), and the life expectancy of people living with HIV (PLHIV) on ART approaches that of the general population[1,2].By 2013, globally there were an estimated 12.9 million PLHIV on ART and 1.1 million lived in South East Asian countries [3].In 2010, the World Health Organization (WHO) recommended the use of better and safer first line ART regimens which include Tenofovir (TDF) and zidovudine (AZT) to avoid the severe and often irreversible side effects caused by stavudine (d4T)- based therapy [4].In the most recent 2013 Consolidated ART guidelines there is furthermore a strong recommendation to phase out d4T in favour of TDF as the latter drug is much better tolerated, has a low frequency of adverse events and can be taken daily allowing a once-a-day fixed-dose combination of tenofovir-lamivudine-efavirenz for treatment[5].

While TDF is well accepted by physicians and patients, one principal concern is renal toxicity. A systematic review and meta-analysis of 17 studies indicated that TDF was associated with a greater loss of kidney function (as measured by creatinine clearance) and a greater risk of acute renal failure compared with control subjects [6]. However, these effects are thought to be modest, and do not preclude the use of TDF in resource-poor areas where monitoring of renal function is not feasible. WHO recommends the need for further research in this area to better understand first, the magnitude of long-term toxicity, and second, whether there are any pre-existing factors for the development of renal dysfunction such as age, hypertension, diabetes and concomitant use of potentially nephrotoxic drugs [5].This information will be helpful in determining whether laboratory screening and monitoring of renal function in resource-poor settings should be routine or undertaken only in targeted high risk groups.

Since 2004, Médecins Sans Frontières-Switzerland (MSF-CH) has provided treatment and care for a large cohort of PLHIV in Dawei District in southern Myanmar. Since January 2012, all new patients received a TDF-based ART as a preferred first-line regimen, and since June 2013,in line with WHO guidelines [5],all patients who were previously on a d4T-based regimen have been changed to a TDF-based regimen. All these patients were tested for virological failure by performing viral load tests before switching.As part of routine care for patients on TDF, renal function is monitored by assessing creatinine clearance (CrCl)at base line and regularly during follow-up.

This MSF project in Myanmar provides an opportunity to measure incidence and risk factors for renal dysfunction in a real world setting in a large cohort of PLHIV on TDF-based ART with serial serum creatinine measurements and CrCl calculations. There are no data in Myanmar on renal dysfunction in PLHIV receiving TDF, so this information will be of value for HIV/AIDS programme management in determining whether renal function monitoring should be recommended in all sites in the country providing TDF-based ART. The aim of this study therefore was to determine the incidence and risk factors for renal dysfunction in PLHIV started on TDF-based ART between January 2012 and December 2013 in Dawei District, Myanmar. Specific objectives were to report on:- i) demographic, clinical and immunological baseline characteristics; ii) the incidence of renal dysfunction after starting TDF in patients with normal renal function at baseline, and their associated risk factors and outcomes; and iii) outcomes of patients with renal dysfunction at baseline.

## Methods

### Study design

This was a retrospective cohort analysis of PLHIV who started TDF-based first-line ART regimen

### Study setting

#### HIV treatment and care in Myanmar

Myanmar is located in Southeast Asia with an estimated population of 51.4 million, of whom 60% live in rural areas [7].Administratively, the country is divided into 14 states/regions, 65 districts and 325 townships. In 2012, the overall HIV prevalence was 0.5%, with higher prevalence in key populations-6% in female sex workers, 7% in men who have sex with men and 17% in injecting drug users [8].National ART scale up started in 2010, and by the end of 2013, over 67,000 people were on ART[9].

The criteria for starting ART in PLHIV are CD4 count less than 350copies/ml or WHO clinical stage 3 or 4, in line with the WHO 2010 Guidelines [4].An estimated 40% of PLHIV are provided with ART through the public sector under the National AIDS Program (NAP), and the remainder is provided by private clinics, and international and local non-governmental organizations. Before 2010, d4T-based ART was the most commonly used first line ART regimen in the country. The National Guidelines for ART in 2010 recommended either AZT-based or TDF-based ART regimen as preferred first line regimen to avoid the adverse effects of d4T[10]. The National Guidelines also recommended screening of PLHIV on TDF for renal dysfunction by assessing CrCl. However the implementation of renal function monitoring is sub-optimal in the country due to lack of resources, and there are no specific recommendations on timing or frequency of conducting tests.

#### HIV treatment and care in MSF project

MSF-CH HIV care and treatment clinic is located in Dawei District, Tanintharyi region in southern part of Myanmar since 2004. Patients receiving care and treatment in this clinic are from Tanintharyi regions and nearby states. In the MSF-CH clinic, there were over 3000 patients who were on first line and second line ART by the end of 2013, treated according to the national protocol. About 50% of patients on first line ART were on TDF-based regimen and the rest of the patients were on AZT, d4T or Abacavir-based regimens. Baseline CrCl is measured in all patients about to start TDF-based ART. TDF is initiated for those with baseline CrCl≥50ml/min/1.73m^2^.Those with CrCl<50ml/min/1.73m^2^ are supposed to start on non-TDF-based ART, although exceptions are made by clinicians. PLHIV are also screened for diabetes, hypertension, and hepatitis B(with the use of the Determine HBsAg test, Alere Medical Co., Ltd., Chiba, Japan) and hepatitis C (with the use of the OraQuick HCV Rapid Antibody Test, OraSure Technologies, Inc., Pennsylvania, USA); and information on use of nephrotoxic drugs and underlying renal disease (from patient records or previous abnormal renal function tests) is also recorded. Diabetes is diagnosed if the Random Plasma Glucose is> 11.1mmol/l or Fasting Plasma Glucoseis > 7.0 mmol/l. Hypertension is diagnosed if the systolic blood pressure is ≥160 mmHg and/or diastolic blood pressure is ≥90 mmHg at two visits within three months. The drugs used to manage hypertension include hydrochlorothiazide, enalapril, amlodipine and atenolol while those used to manage diabetes include metformin and glibenclamide.

After starting TDF, CrCl is calculated at 6-monthly intervals. Patients with baseline risk factors such as chronic renal disease, diabetes, hypertension, hepatitis B and hepatitis C are screened at 1 month and 3 months after initiation, then every six months. All treatment and laboratory investigations are provided free of cost.

#### Definition for renal dysfunction

We defined renal dysfunction as CrCl less than 50 ml/min/1.73m^2^ and severe renal dysfunction as CrCl less than 30 ml/min/1.73m^2^.Serum creatinine measurements were performed at the clinic laboratory using rapid test (StatSensor, Nova Biomedical Corporation, Massachusetts, USA), and CrCl is calculated by using the Crockcroft-Gault formula recommended by WHO[5].

### Study population

All adults (aged 15 years or above) with HIV infection who started on first-line TDF-based ART between January 2012 and December 2013and followed for a period of at least 6 months were included in the study.

### Data variables

Data variables included: age, sex, previous ART exposure, ART start date, other medication (at start and during ART), co-morbidities (at start and during ART), results of hepatitis B and C testing, height, weight, WHO clinical stage, CD4 cell count, CrCl (at start and during ART along with the date) and patient outcomes. These data were collected from an electronic FUCHIA database(Follow Up and Care for HIV Infection and AIDS)(2010 Epicentre, http://www.epicentre.msf.org) and from patient medical files. The electronic database and patient medical files do not include patient names but patient numbers.

### Analysis and statistics

Data were single entered and analysed for frequencies and proportions in EpiDataversion 3.1 for data entry, and version 2.2.2.182 for analysis [11].The records were anonymized and de-identified prior to analysis.Data were censored on July 1, 2014. Death and loss to follow up patients were also censored and this was taken account of in the Kaplan Meier plots. In PLHIV with normal renal function at baseline, time to event analysis was carried out using STATA version 12.1and a Kaplan Meier plot used to estimate cumulative incidence of renal dysfunction. Unadjusted Cox proportional hazard ratios were calculated to assess the possible association of characteristics with the development of renal dysfunction. Characteristics at a threshold *P* value of <0.2 in the univariate analysis were included in the multivariate Cox regression model and adjusted hazard ratios with 95% confidence intervals were calculated. A *P*-value of less than 0.05 was considered statistically significant.

### Ethics

The study was approved by the Ethics Advisory Group of the International Union Against Tuberculosis and Lung Disease, Paris, France, and the Ethical Review Committee of the Department of Medical Research (Lower Myanmar). As the study only involved a review of patient records, the need for informed patient consent was waived.

## Results

There were 1391 HIV-infected patients started on a first-line TDF-based ART regimen during the study period and the median duration (IQR) of follow up was 1.14 (0.78–1.6) years. Their median (interquartile range) age was 38(33–42) years. Demographic, clinical and immunological baseline characteristics are shown in **Table 1.** 58% of patients had previously been on other ART regimens, either D4T-based (N = 749, 54%) or AZT-based ART (N = 56, 4%). Over 90% of patients were taking concomitant cotrimoxazole preventive therapy. Less than 10% had additional co-morbidities such as diabetes, hypertension or pre-existing renal disease. Of those tested for Hepatitis B and Hepatitis C, 11% and 16% respectively were positive. 20% of patients were underweight (BMI<18.5). In terms of immune suppression, 83% of patients were in WHO Clinical Stage 3 or 4 and 32% had a CD4 cell count <200/mm^3^. There were 19 (1.3%) patients who had renal dysfunction at the start of TDF-based ART, all of whom had a CrCl between 30 and 49 ml/min/1.73m^2^. During follow-up, few patients were newly diagnosed with diabetes or hypertension during ART, and 3% were given potentially nephrotoxic drugs (see **Table 2**).

**Table 1:** Baseline characteristics of HIV-infected patients started on tenofovir-based antiretroviral therapy between January 2012 and December 2013 in Myanmar.

Baseline characteristics		Number	Percentage
Total	All patients	1391	100
Age group in years	15–24	47	3
	25–44	1090	79
	≥45	254	18
Gender	Male	566	41
	Female	825	59
Medication	Previous ART Exposure	805	58
	Concomitant use of cotrimoxazole	1313	94
Co-morbidities	Diabetes Mellitus (already known)	16	1
	Hypertension (already known)	116	8
	Renal disease (already known)	9	<1
Hepatitis B	Tested:	1152	83
	Hepatitis B surface antigen positive	132	11 ^a^
Hepatitis C	Tested:	444	32
	Hepatitis C antibody positive	69	16 ^a^
BMI (kg/m^2^)	<16.0	57	4
	16–16.9	55	4
	17.0–18.4	171	12
	18.5–24.9	128	9
	25 and above	980	71
WHO Clinical Stage	1	70	5
	2	166	12
	3	719	52
	4	436	31
CD4 Cells/mm^3^	<200	441	32
	200 and above	935	67
	Not recorded	15	1
Renal status (baseline)	Normal renal function ^b^	1372	99
	Renal dysfunction ^c^	19	1

BMI = body mass index; WHO = World Health Organization

^a^ Percentage of those tested

^b^ Creatinine clearance ≥ 50ml/min/1.73m^2^

^c^ Creatinine clearance < 50ml/min/1.73m^2^

**Table 2:** Diseases that were newly diagnosed or nephrotoxic drugs that were given in HIV-infected patients with normal renal function on tenofovir-based antiretroviral therapy between January 2012 and December 2013 in Myanmar.

Events that occurred after start of ART	Number	Percentage
All patients	1372	100
Diabetes Mellitus	18	1.3
Hypertension	23	1.7
Use of nephrotoxic drugs^a^	37	3

ART = antiretroviral therapy

^a^ included amphotericin B, amikacin, acyclovir, non-steroidal anti-inflammatory drugs

**Fig 1:**
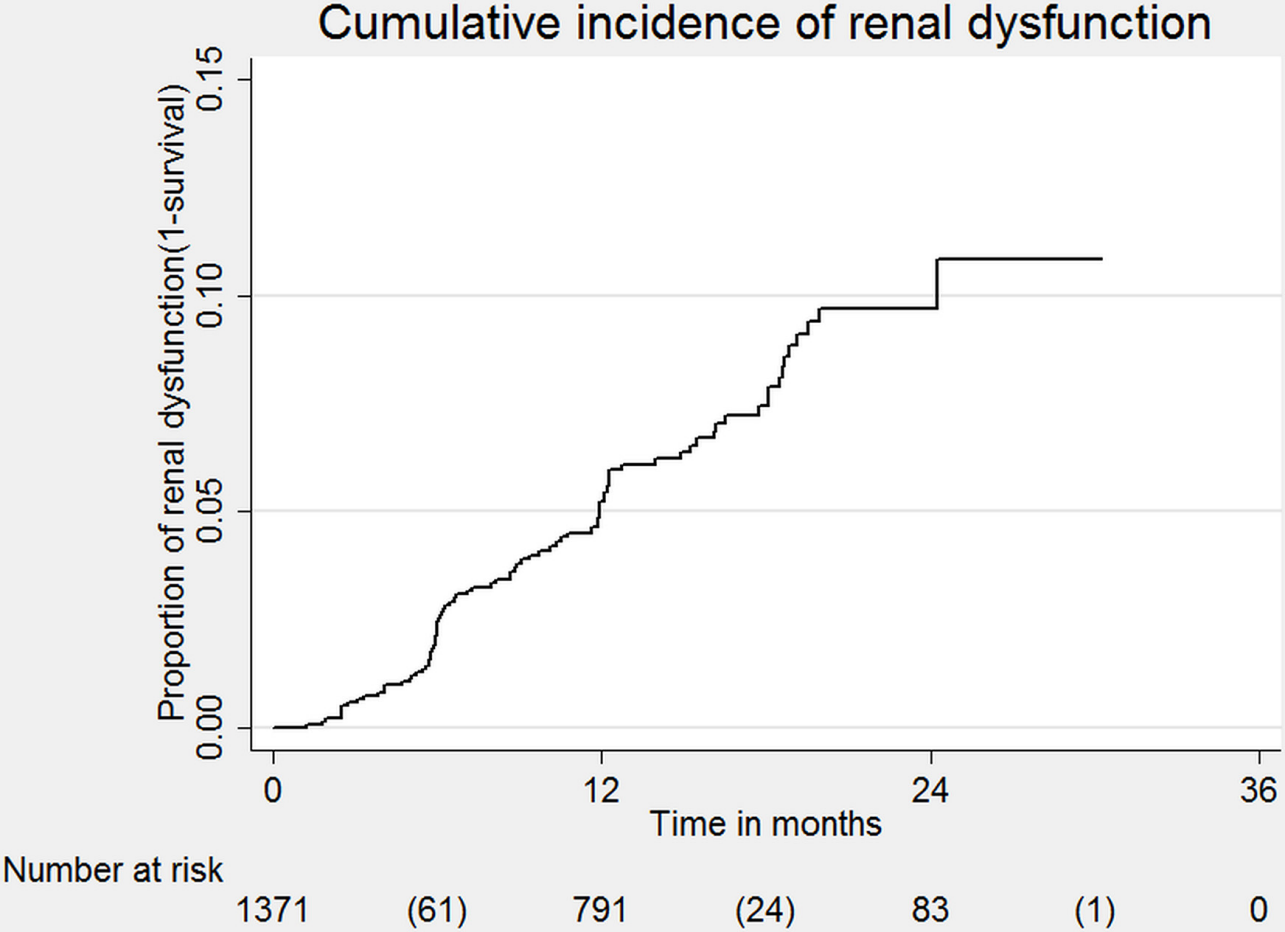
Cumulative incidence of renal dysfunction in HIV-infected patients

Of 1372 patients with normal baseline renal function who were followed for a total of 1632 person-years, 86 (6.3%) developed renal dysfunction, 79(5.8%) died and 39(2.8%) were lost to follow up during the study period. The incidence rate of renal dysfunction was 5.4 per 100 person-years of follow-up. The Kaplan-Meier curve shows the cumulative incidence of renal dysfunction from the time when patients started treatment with TDF. The cumulative incidence of patients developing renal dysfunction over time was 4% at 12 month and 8% at 24 months from the time of TDF initiation (**Fig 1**).Of 86 patients who developed renal dysfunction, 78 had creatinine clearance between 30–50ml/min/1.73m^2^ and were maintained on TDF–based ART by the end of follow-up. Of the remainder, two patients died, one was transferred out, four were changed to a non-TDF ART regimen due to CrCl<30ml/min/1.73m^2^, and one was changed to a non-TDF containing second line regimen due to first line ART failure. Risk factors associated with the development of renal dysfunction are shown in **Table 3**. In the adjusted analysis, female gender, age ≥45 years, diabetes, renal disease, under-nutrition, CD4 count <200cells/mm^3^were associated with developing renal dysfunction.

**Table 3:** Characteristics at baseline and during treatment in HIV-infected patients with normal renal function on tenofovir-based ART who developed renal dysfunction during follow-up in Myanmar.

Characteristics		Renal Dysfunction N (%)	Rate per 100 person yrs	Unadjusted HR^§^(95% CI)	*P* value	Adjusted HR^§^(95% CI)	*P* value
Total		86(6)	5.4				
Age group in years	15–24	0	-	-	-	-	-
	25–44	47 (4)	3.7	Ref		Ref	
	≥45	39(16)	13.9	3.8 (2.5–5.8)	<0.001	3.4 (2.2–5.2)	<0.001
Gender	Male	38(5)	4.1	Ref		Ref	
	Female	48(9)	7.4	1.8 (1.2–2.8)	0.01	1.8 (1.2–2.9)	0.009
Previous ART	Yes	41(5)	4.3	0.6 (0.4–0.9)	0.02	1.1(0.6–1.9)	0.8
	No	45(8)	7.1	Ref		Ref	
Use of cotrimoxazole	Yes	44(8)	7.2	1.7 (1.1–2.6)	0.02	1.2 (0.7–1.9)	0.51
	No	42 (5)	4.3	Ref		Ref	
Nephrotoxic drugs	Yes	5(14)	12.6	2.4 (1.0–6.0)	0.10	2.1(0.8–5.4)	0.11
	No	81(6)	5.2	Ref		Ref	
Diabetes Mellitus^a^	Yes	7 (21)	19.1	3.8 (1.7–8.2)	<0.001	3.6 (1.6–8.2)	0.002
	No	79(6)	5.1	Ref		Ref	
Hypertension^b^	Yes	13(10)	8.5	1.7 (0.9–3.1)	0.08	1.4 (0.7–2.6)	0.34
	No	73(6)	5.1	Ref		Ref	
Known Renal Disease	Yes	3(33)	27.9	5.4 (1.7–17)	0.004	3.5 (1.1–11.4)	0.04
	No	79(6)	5.1	Ref		Ref	
	Unknown	4(17)	15.1	3 (1.1–8.1)	0.03	3.6 (1.3–10.5)	0.02
Hepatitis B	Negative	67(6)	5.2	Ref			
	Positive	13(9)	7.2	1.4 (0.8–2.5)	0.26		
	Not tested	6(6)	5.1	1.0 (0.4–2.2)	0.94		
Hepatitis C:	Negative	26(7)	5.5	Ref			
	Positive	2(3)	2.1	0.4(0.1–1.6)	0.20		
	Not tested	58(6)	5.7	1.0 (0.7–1.6)	0.89		
BMI (kg/m^2^):	<16.0	9(16)	17.3	3.9 (1.9–7.9)	<0.001	2.3 (1.1–5)	0.03
	16–18.5	24(10.8)	10.0	2.3(1.4–3.7)	0.17	1.8 (1.1–3.2)	0.02
	18.5–24.9	52(5.4)	4.5	Ref		Ref	
	25 and above	1(0.8)	0.7	0.2(0.0–1.1)	0.06	0.2(0.0–0.9)-	
WHO Clinical Stage:	1	4(6)	5.4	Ref		-	-
	2	7(4)	3.8	0.7 (0.2–2.)	0.58	-	-
	3	46(6)	5.4	1.0 (0.4–2.8)	1.0	-	-
	4	29(7)	6.0	1.1 (0.4–3.2)	0.84	-	-
CD4 Cell /cumm^3^	<200	43(10)	9.1	2.6 (1.7–4.0)	<0.01	2.3 (1.2–4.3)	0.02
	200 and above	39(4)	3.6	Ref			
	Not recorded	4(27)	20.3	5.9(2.1–16.6)	<0.01	1.9 (0.6–5.8)	0.32

ART = antiretroviral therapy; HR = hazard ratio; CI = confidence interval; BMI = body mass index; WHO = World Health Organization

^a^ included patients who had diagnosed diabetes mellitus before or after TDF started

^b^ included patients who had diagnosed hypertension before or after TDF started

^§^Unadjusted and adjusted HR were calculated by using Cox proportional Hazard methodology

Of 19 (1%) patients who had renal dysfunction at the start of TDF-based ART, all continued on the TDF regimen: CrCl remained between 30–49 ml/min/1.73m^2^ in five patients and the remainder regained normal renal function.

## Discussions

To the best of our knowledge, this is the first study in Myanmar to assess renal function in patients starting on TDF-based ART and the incidence of renal dysfunction during follow-up. The majority of patients had normal renal function at baseline. Just over six percent of these patients developed renal dysfunction during follow up, which is similar to recent studies done in Asia [12,13], but in the majority of these patients this was not severe enough to warrant a change in therapy. Only four patients (0.3%) progressed to severe renal dysfunction needing a change to an alternative ART regimen, with these findings mirroring those from elsewhere[14].These overall results are in line with previous studies which concluded that the clinical magnitude of renal toxicity during TDF-based ART is modest[6].This low incidence of renal toxicity is further confirmed by more recent publications from Africa and Asia [15,16]although differences in definitions of renal dysfunction and duration on TDF-based ART make it difficult to compare findings from one study to another.

We found that baseline factors such as age≥45 years, being female, underweight, having underlying renal disease and diabetes, and low CD4 count were associated with the development of renal dysfunction. These findings are similar to those of other studies. Women with low body weight are at risk of renal toxicity due to exposure to high TDF drug concentrations [17].Older age, comorbidities with diabetes, lower BMI and low CD4 count are also documented as risk factors in studies from Africa and Asia [18,19].However, these findings are not consistent as one report from USA showed no association with pre-existing renal risk factors [20].

There were a few patients (1%) with pre-existing renal dysfunction who were started on TDF that was not in accordance with protocol. Reassuringly, most of these patients regained normal renal function during follow-up and in the remainder none progressed to severe renal disease. HIV infection itself is a cause of renal disease[21]and our findings suggest that TDF-based ART is an acceptable regimen for treating such patients. Improvement in renal function in those with pre-existing renal dysfunction has previously been recorded [16,22].

The strengths of this study were the large cohort of patients registered and followed within a routine programme setting. As a result of reasonable laboratory infrastructure, we were also able to screen for co-morbidities such as diabetes and assess for the presence of hepatitis B and C infection. Since the proportions of deaths and lost to follow up during study period were low, outcomes were less likely to be affected. We used STROBE guidelines and sound ethics principles for the conduct and reporting of this observational study [23,24]. There were some limitations. First, we used the Cockcroft Gault formula to calculate CrCl to estimate glomerular filtration rate, but other serum creatinine-based estimation equation such as Modification of Diet in Renal Disease formula was shown to have higher accuracy to estimate CrCl in HIV infected adult [25]. Second, the main targets of TDF renal toxicity are the proximal renal tubules but we did not assess renal tubular dysfunction because of the need for additional laboratory analysis which was unavailable during the study period[26].Third, the data were collected retrospectively from patient records which may have had inaccuracies.

This study strengthens the evidence base for the country wide management of HIV-infected patients starting TDF-based ART. The low incidence of renal toxicity suggests that routine assessment of CrCl may not be necessary. However, care needs to be taken with certain high risk groups such as those aged ≥45 years and those with underlying renal disease, diabetes, underweight and severe immune suppression, and if resources permit, they should have creatinine clearance measured at baseline and during follow up. Further work is needed to a) monitor renal function for longer periods of time in order to assess longer term outcomes, and b) understand the magnitude, relevance and programmatic importance of renal tubular toxicity.

In conclusion, this study on nearly 1400 HIV infected patients starting first line TDF based ART has shown a low incidence of renal dysfunction during follow up. Patients with certain characteristics such as age ≥45 years, underlying renal disease, diabetes, underweight and severe immune suppression—are at increased risk of renal dysfunction, and may require targeted renal monitoring during treatment if resources permit.

## Supporting Information

S1 Dataset Renal dysfunction and TDF regimen, Myanmar. (XLSX)
